# Supplementary material for: The causal relationship between the human gut microbiota and pyogenic arthritis: a Mendelian randomization study
Source: Front Cell Infect Microbiol. 2024 Nov 26;14:1452480. doi: 10.3389/fcimb.2024.1452480 (PMC11629706; doi:10.3389/fcimb.2024.1452480)
Supplement: Supplementary file 1 [file DataSheet1.zip › mendelian test/ebi-a-GCST90027514.csv_forest.pdf]

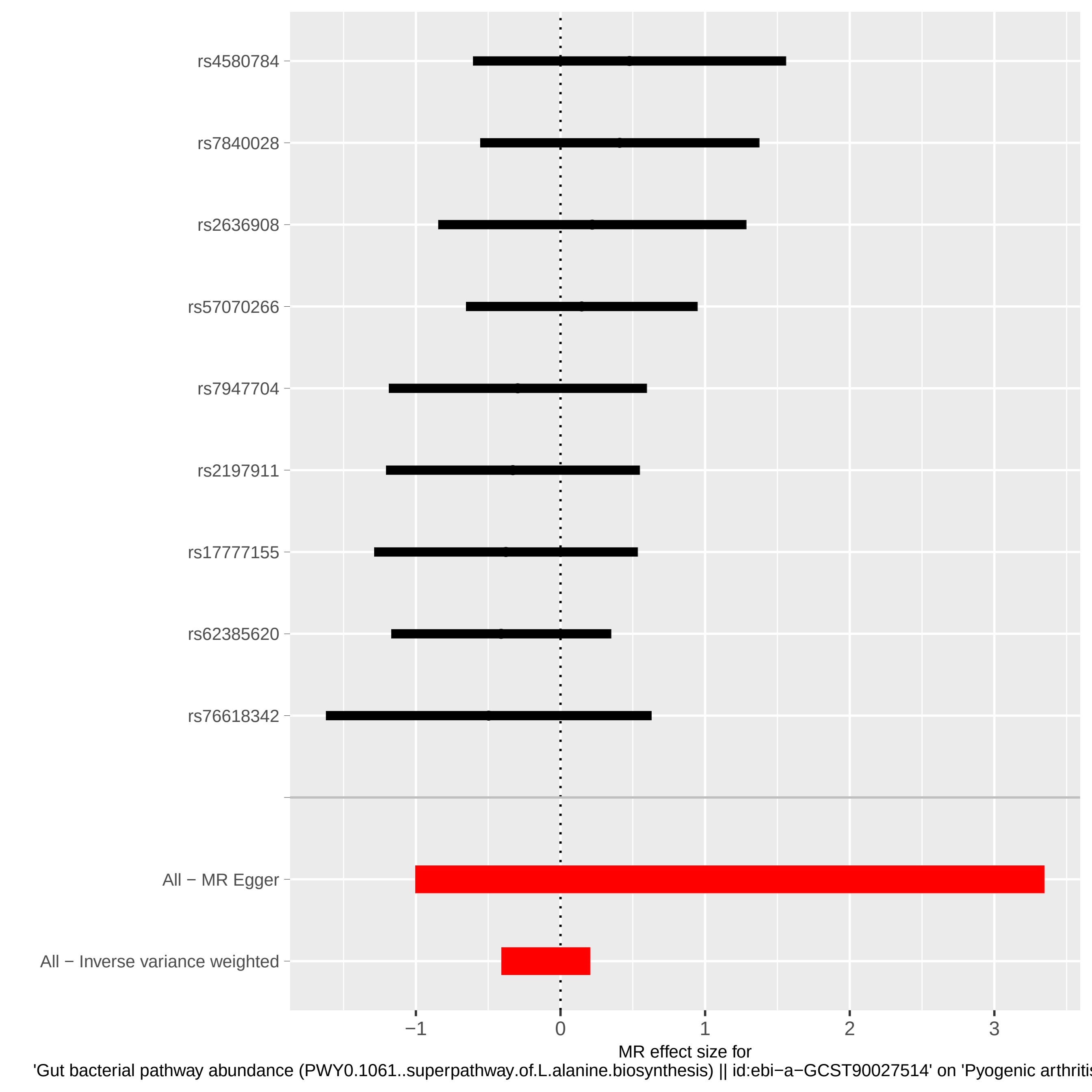

rs4580784
rs7840028
rs2636908
rs57070266
rs7947704
rs2197911
rs17777155
rs62385620
rs76618342
All − MR Egger
All − Inverse variance weighted
−1
0
1
2
3
MR effect size for
'Gut bacterial pathway abundance (PWY0.1061..superpathway.of.L.alanine.biosynthesis) || id:ebi−a−GCST90027514' on 'Pyogenic arthriti
